# The possibility of establishing causes of death on the basis of the exhumed remains of prisoners executed during the communist regime in Poland: the exhumations at Powązki Military Cemetery in Warsaw

**DOI:** 10.1007/s00414-014-1084-z

**Published:** 2014-09-17

**Authors:** Łukasz Szleszkowski, Agata Thannhäuser, Krzysztof Szwagrzyk, Tomasz Jurek

**Affiliations:** 1 Department of Forensic Medicine, Medical University, ul. Mikulicza-Radeckiego 4, 50-386 Wroclaw, Poland; 2 The Institute of National Remembrance, Poland, ul. Sołtysowicka 21a, 51-168 Wroclaw, Poland

**Keywords:** Forensic pathology, Exhumation, Capital punishment, Judicial hanging, Gunshot to the head, Accursed soldiers

## Abstract

This study presents the results of the analysis of forensic examinations of the remains of 194 prisoners exhumed at Powązki Military Cemetery in Warsaw. In all probability, most of those buried there were judicially sentenced to death by firing squad or hanging in connection with activities of the Polish independence underground in its struggles with the postwar communist regime. Forensic medical research focussed on determining causes of death and reconstructing the mechanisms of injury leading to death. Most probable causes of death were found in 108 of 194 cases; of these, 76 were isolated gunshot wounds to the head, mostly directed to the occipital region. In 29 of 194 cases, only extensive skull fractures were observed, making it impossible to determine the mechanism of injury. The condition of these skulls do not permit the exclusion of injuries due to gunshots, which were very likely given the historical context of the studied location. In one case, it is assumed that the cause of death could be blunt force trauma to the head. In 86 of 194 cases, it was not possible to determine the cause of death. Of these cases, 20 skeletons were in such poor condition that erosive changes could have completely obliterated even very extensive head injuries leading to death. No injuries were observed that could be associated with execution by hanging.

## Introduction (historical context)

At the end of World War II, Poland found itself in the zone of influence of the USSR. Despite the destruction caused by the war and the huge number of its victims, the postwar years in Poland were a period of continued conflict. Resistance directed against the new Moscow-dependent communist government took the form of both conspiratorial activity and armed (guerilla) combat. Actively operating organisations derived from, among others, the Home Army, which continued its wartime activities [1]. It is estimated that in the period of the greatest terror, i.e. from 1944 to 1956, more than 4000 individuals were executed in Poland on the strength of court sentences, the vast majority of them related to the postwar underground (accursed soldiers). Those executed were buried in secret locations, most of which remain unknown to this day [2]. Among all the correctional facilities in Poland, the prison in Mokotów, in Warsaw, was a special place. Imprisoned in the facility’s ward X, which was managed directly by the Ministry of Public Security, were the most important members of the Polish independence underground. Among them were, for example: Rotamaster Witold Pilecki, who had been the only voluntary inmate of the Auschwitz concentration camp and who operated after the war in the underground Polish Secret Army organisation; General August Emil Fieldorf aka ‘Nil’, one of the chief commanders of the Home Army; and commanders of partisan units such as Major Zygmunt Szendzielarz aka ‘Łupaszko’ or Major Hieronim Dekutowski aka ‘Zapora’. Archival research indicates that in the period 1948–1955, in Powązki Military Cemetery in Warsaw, in an area known as *Łączka* [English, ‘little meadow’], approximately 300 prisoners from Mokotów who had been shot or hanged may have been buried [3]. The search and exhumations being conducted by the Institute of National Remembrance on a wide scale in Poland since 2011 offer a unique opportunity to discover the burial sites and identify and determine the causes of death of the victims of the postwar repression [4].

## Material and methods

**Fig. 1:**
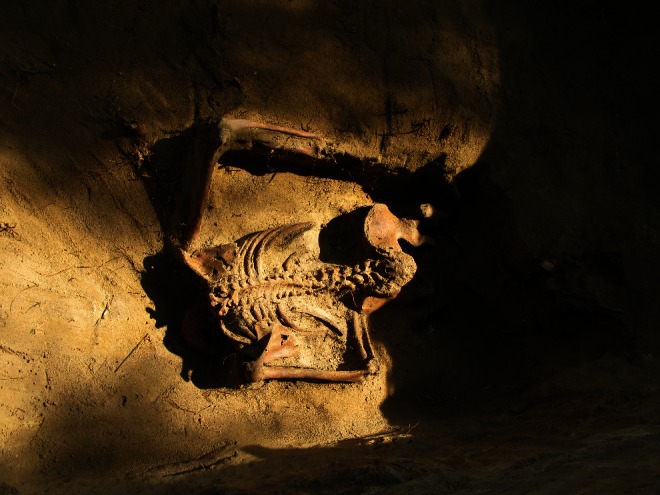
Human remains discovered in a grave pit

The exhumations at Powązki Military Cemetery in Warsaw, the latest study conducted within the framework of the project ‘The search for unknown burial places of victims of communist terror in Poland in the years 1944–56’, were carried out in two stages in 2012 and 2013, on behalf of the Institute of National Remembrance and the Council for the Protection of the Memory and Martyrdom of Independence Fighters. The work was performed by a multidisciplinary team of researchers composed of historians, archaeologists, physical anthropologists, a forensic anthropologist, a forensic pathologist, and forensic geneticists. In total, the remains of 194 individuals were exhumed. These remains were buried in 109 grave pits, both individual and mass, often according to a system deviating from accepted methods of burial (Fig. 1). The exhumations in Warsaw constituted the continuation of a research project carried out in 2011 and 2012 in Wroclaw. The research methodology developed at that time and applied to mass exhumations was used and expanded, in relation to both archaeological work and forensic medical examination of the remains [5, 6]. In each case, researchers from the Department of Forensic Medicine of the Medical University of Wroclaw conducted a full forensic medical and anthropological examination of the remains in the examining field laboratory, located in the cemetery in the immediate vicinity of the exhumation. After arrangement of the remains in anatomical order, their state of preservation was described, including erosional changes. Morphological features useful in establishing a biological profile, individual identifying features, disease-induced changes and wounds, were reported in detail. In each case, material was secured for genetic identification tests conducted at the Pomeranian Medical University in Szczecin by the Polish Genetic Base of the Victims of Totalitarianism. Forensic medical examination of the remains, apart from gathering identifying data [7], focussed on finding the most probable cause of death along with the mechanism of injury which led to it. Traumatic changes to the bone were reported in the examining protocol, following reconstruction (gluing of fragments) of damaged bone elements (especially in cases of comminuted fractures of the skull). In addition, extensive photographic documentation of wounds was prepared, as well as freehand sketches. Descriptions and assessments of the nature of bone injuries and conclusions regarding the mechanism of injury and the probable causes of death were made by the forensic pathologist and were based on a standard procedure used in forensic medical practice [8–10]. In analysing the cause of death, a small number of changes that could not (due to erosional changes) be clearly classified as perimortem trauma were not taken into account.

## Results

**Fig. 2:**
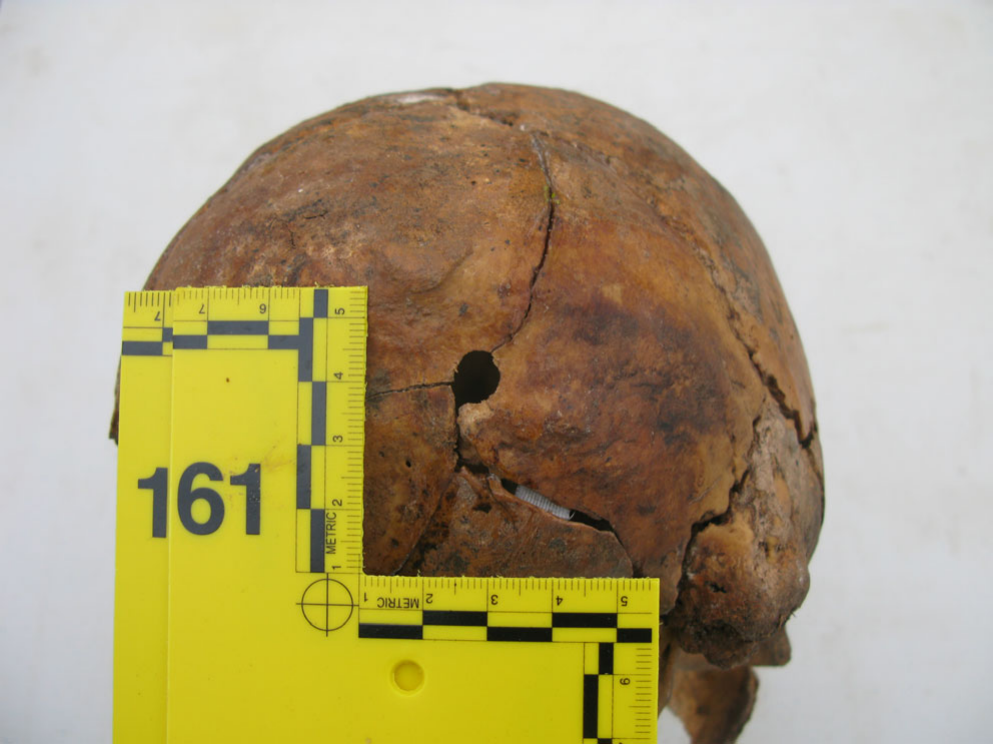
Entrance wound in a squamous portion of occipital bone

**Fig. 3:**
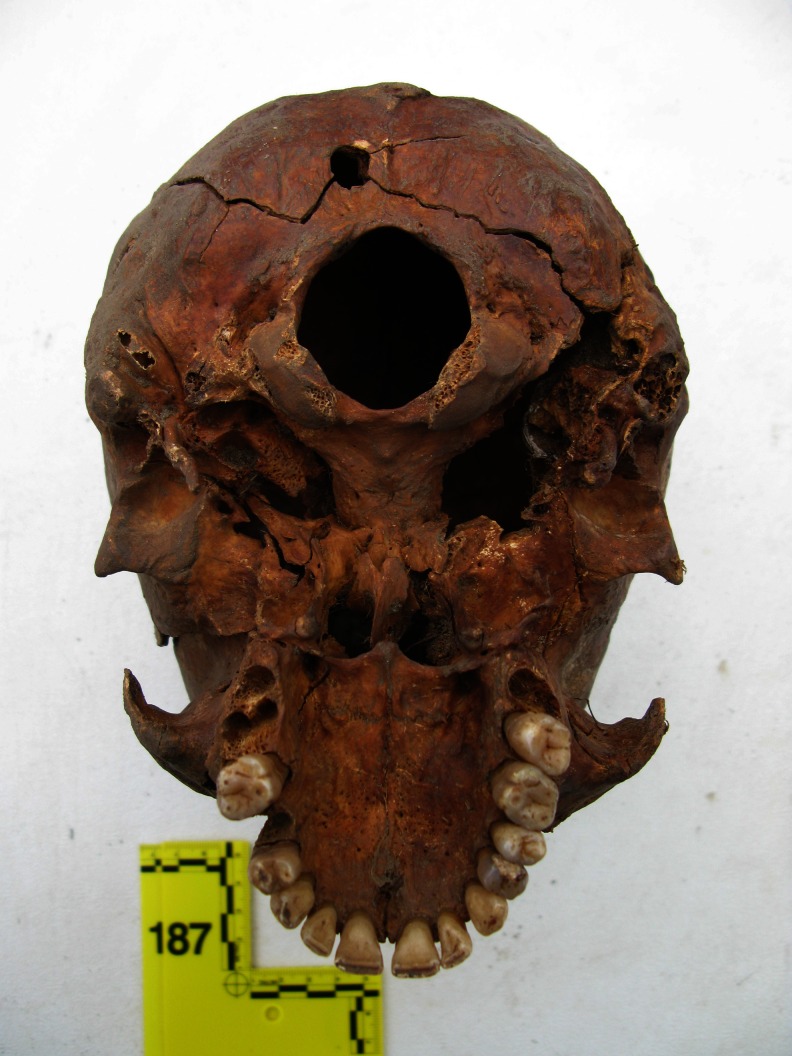
Entrance wound in a squamous portion of occipital bone

**Fig. 4:**
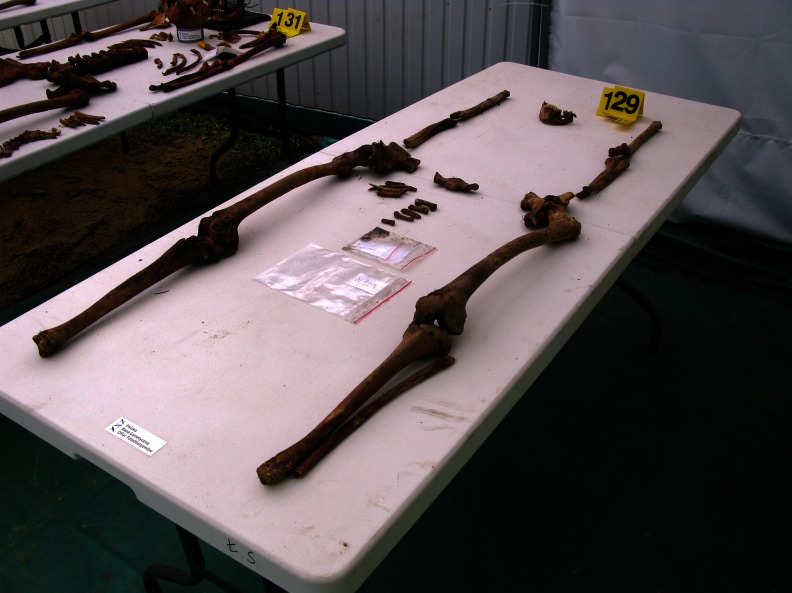
Remains subjected to severe erosional changes

In 108 of 194 cases, forensic medical examination of the skeleton enabled us to determine the most probable cause of death (Table 1). In 78 of 194 cases, the presence of a gunshot wound to the bone was established. Most of these cases (*n* = 76) involved an isolated gunshot wound to the skull. In 19 of 78 shooting cases, both entrance and exit wounds (or fragments thereof) were isolated, whereas in 41 of 78 cases, only the entrance wound (or fragments thereof) was identified. In the vast majority, i.e. 56 of 60 cases, they were located in the rear area of the skull (Figs. 2 and 3). However, in 18 of 78 cases, only exit wounds (or fragments thereof), most often located in the front part of the skull (*n = 35*/*37*), were isolated. The locations of entrance and exit wounds are shown in Tables 2 and 3. In 5 of 76 cases, multiple gunshot wounds to the head (double) were identified; in the remaining cases, single gunshots. In no case were gunshot injuries located exclusively in the postcranial skeleton; however, in 2 of 78 cases, gunshot wounds of the head were accompanied by such injuries. The first of these involved a shot to the head, with an entrance in the occipital region, with accompanying gunshot damage to the left humerus and two ribs on the left side. The second case, involves three shots to the head, with 11 bullets present in the grave pit, mostly located in the area of the torso, including the spinal vertebrae and a gunshot to the proximal phalanx of the left great toe. The locations of the entrance wounds on the skull in this case were atypical, i.e. in the right temporal-parietal region, with the path of the bullet running steeply downwards. Gunshot damage to the vertebrae could be determined only during in situ examination of the skeleton in the grave pit. After extraction of the skeleton from the grave pit, the bony elements of the vertebrae disintegrated completely, making it impossible to carry out inspection and documentation of injuries in the examining laboratory. In 29 of 194 cases, the presence of extensive and comminuted fractures of the skull was noted. In 9 of the 29, the pattern of fissured fractures (including radial and converging, often in a location and pattern analogous to those found in cases of gunshot injuries) indicated a high probability of a gunshot wound of the head, despite the failure to observe entrance or exit defects. In the remaining cases, due to missing bone fragments, it was impossible to determine the mechanism of injury. In five cases, the most severe bone fractures were located at the base of the skull, with fissures running along the cranial vault. Due to the condition of the remains, possible gunshot wounds to the bone, which might have been obliterated by erosive changes involving bone fragments, could not be ruled out in any of these cases. To sum up, the presence of extensive head wounds (confirmed gunshot wounds as well as those caused by an undetermined mechanism) was declared in a total of 107 of 194 cases. In one case only, it was found that the most probable cause of death was blunt force trauma (BFT) to the head. In this case, involving a completely preserved skull, which bore traces of having been autopsied, a single fissure fracture of the cranial vault and base was noted. In 86 of 194 cases (ca 44 %), it was impossible to determine the most probable cause of death. Among these, in 20 cases, severe erosive changes involving the skeletons prevented exclusion of even very extensive gunshot wounds to the head, which constituted such a large percentage of cases in the studied material. In the cases of these skeletons, only fragmentary long bones (sometimes only portions) and small fragments of other bones were preserved (Fig. 4). In 7 of 86 cases, the preserved fragmented skulls revealed the presence of single, mostly short fissure fractures, possibly the result of perimortem trauma. Due to the poor condition of the skulls and the limited extent of the preserved fractures, there were no grounds for assuming that they represented the most probable cause of death.

**Table 1:** Most probable cause of death

Most probable cause of death	Number of cases (*n* = 194)
Shot to the head	76
Shot to the head and torso	2
Extensive head injuries probably due to gunshot	9
Extensive head injuries, mechanism unknown	20
Blunt force trauma (BFT) to the head	1
Cause of death undetermined	86

**Table 2:** Entrance wounds

Gunshot wounds in the skull (*n* = 78)	
Location of entrance wound	Number of cases (*n*)
Squamous portion of occipital bone	47
Squamous portion of occipital bone in the vicinity of the foramen magnum	6
Squamous portion of occipital bone in the vicinity of the foramen magnum with damage to the C1/C2 vertebrae	3
Frontal squama	2
Parietal bone	2

**Table 3:** Exit wounds

Gunshot wounds in the skull (*n* = 78)	
Location of exit wound	Number of cases *(n)*
Frontal squama	23
Front part of the parietal bone	6
Frontal-parietal region	2
Mandible	4
Rear part of the parietal bone	1
Temporal bone	1

## Discussion

The exhumations of victims of the communist regime at Powązki Military Cemetery in Warsaw represent the latest mass exhumations carried out in Poland in the years 2011–2013. In addition to identification studies, the cause of death and mechanism of injury were determined and the method of execution reconstructed [5, 6, 11]. In the course of the studies, in each case where the most probable cause of death was established, the wounds were classified as ‘lethal’; that is to say, it was assumed that had they been sustained during life and they would have led to death within a short period of time, due to damage to vital organs [12]. Contrastingly, there were no cases of the type defined by Baraybar and Gasior as ‘lethal if untreated’, e.g. isolated skeletal injuries suggestive of gunshot wounds to the abdomen or proximal limbs [12]. These results appear understandable in light of the fact that the victims were executed individually on the basis of a court sentence. The execution had to result in the death of the convict, confirmed by a doctor; hence, the method of execution (shot to the head) had to be effective and cause rapid death. Injuries of a lethal if untreated nature would appear to be more characteristic of the victims of mass executions, battles, skirmishes or accidental shootings. Cases of extensive head injuries found during the exhumations in Warsaw may also be the result of execution by firing squad. However, due to erosional changes, it is impossible to either confirm or rule out gunshots as the mechanism of injury, though they are very likely given the historical context of the study location. Archival data on prisoners buried there indicate that the majority (about 85 %) were executed by firing squad. The results of research conducted at Powązki Military Cemetery in Warsaw are consistent with the results of research conducted during the exhumations at Osobowicki Cemetery in Wroclaw in 2011–2012 [6, 11]. Shots to the back of the head also dominated, although there was a greater variety of gunshot wounds (e.g. to the postcranial skeleton). The gunshot wounds observed in the skulls are clearly analogous with the results of the work carried out by Polish forensic pathologists in the burial sites of Polish officers in Katyn, Kharkov and Mednoye. Near these locations, in 1940, more than 10,000 Polish officers, prisoners of war held in camps in Kozelsk, Starobilsk and Ostashkov, were murdered by NKVD officers [13]. In the case of exhumations in Katyn, gunshot wounds to the head were found in 77.8 % of the exhumed skeletons, of which approximately 97 % were shots to the occiput [14]. During exhumations in Kharkov, gunshot wounds were found in 39 % of the exhumed skeletons, of which 73 % were wounds of the occiput [15], whereas in Mednoye, gunshot wounds to the head were found in 74 % of the cases, with a clear predominance of shots to the occiput [16]. Irrespective of the relatively small differences in the location of the entrance and exit defects in the skulls, the authors of this publication use the term ‘Katyn method of execution’ for shots to the rear of the head, fired from small arms at close range. However, as is apparent from these studies of the exhumation of prisoners of the camps of the former USSR, in some cases (from 22–61 %), it was not possible to determine the cause of death, even though the archival data suggest a very high probability that all the officers were killed via a similar mechanism of injury, i.e. from a shot to the head [14–16]. The authors of these studies explain this in terms of the condition of the skeletons (fragmentation, incompleteness, erosional processes, the possibility of gunshots to the nape of the neck), which is also consistent with the findings resulting from examination of the remains exhumed in Warsaw. Archival data indicate that about 15 % of inmates of the Warsaw prison who could have been there between 1948 and 1955 and who were buried in Powązki were executed by hanging. However, in no case was it possible to confirm the presence of cervical spine fractures which could be associated with execution by hanging [17]. In such executions, the presence of changes of this kind depends on, among other factors, methods of attaching the noose and of carrying out the execution (use of a trapdoor, length of the rope) [17]. Cervical spine fractures are extremely rare in typical hanging cases; fractures of the thyroid cartilage and hyoid bone are likewise infrequent. Retrospective studies by Feigin give the incidence of all fractures of various parts of the neck (spine, hyoid bone and thyroid cartilage) as about 9.5 %, whereas fractures of the cervical spine in the studied remains accounted for less than 1 % [18]. In about half the cases, the action of the noose around the neck is unaccompanied by even soft tissue damage [17]. Research on the exhumed remains of prisoners sentenced to death by hanging using the trapdoor, with the knot of the noose located under the left ear, showed that fracture ‘was not usual’ [19]. Therefore, confirmation of hanging as the cause of death, based on the study of exhumed skeletal remains of individuals executed more than 60 years earlier, should be considered very unlikely to the point of being unique. Moreover, in the research conducted in Osobowicki Cemetery in Wroclaw in 2011–2012, the presence of such injuries was not revealed in any case. In analysing the cases in which forensic medical examination failed to determine the cause of death, several possible factors should be considered. Erosional changes could erase all traces of injuries, even as extensive as gunshot wounds to the head, from bones. Fatal wounds could affect the soft tissues (internal organs) while leaving no traces on the bones. Death could also be a consequence of changes due to disease, which would leave no traces on the skeleton. In the overall analysis of the data, one more very important factor should be taken into account, namely, the incompleteness of archival data and hence a certain degree of difficulty in foreseeing potential problem areas associated with the studied location (the place where prisoners were buried). From the archival data, it appears that prisoners who died from causes other than execution, for reasons such as illness, or even prisoners from other prisons, might happen to be buried in Powązki [20]. The incomplete state of archival data appears to be one of the main concerns, also emphasised by other authors [21], associated with the exhumation of victims from several decades past, a concern which should also be taken into account when considering the possible cause of death. Therefore, the authors advocate extreme caution in the interpretation of all data collected in the course of archival, archaeological and forensic studies of victims of the communist regime, both in Poland and in other countries of the former Eastern bloc.

## Conclusion

In a study of skeletal remains that have lain for decades in the earth, it is not possible, due to the absence of soft tissue, to determine an indisputable cause of death. In the course of these studies, the presence of erosive changes, including those affecting bones, can obscure even such extensive injuries as gunshot wounds to bones of the skull. Despite archival data indicating the possibility of execution by gunshot, after so many years, it is no longer possible to determine the most probable cause of death in each case of execution. In cases where the condition of the skeleton permits forensic conclusions, it can be stated that the cause of death of the prisoners sentenced to death from the Warsaw prison was a gunshot to the head, particularly the occiput, which corresponds to similar research carried out in other parts of Poland as well as the burial sites of Polish officers and inmates of POW camps in the former USSR executed in 1940 by the NKVD.
